# Exploring a Rare Association: Systematic Review of Hypercalcemia in Nontuberculous Mycobacterial Infections

**DOI:** 10.3390/microorganisms13040773

**Published:** 2025-03-28

**Authors:** Ramon Cohen, Viviana Ostrovsky, Lior Zornitzki, Daniel Elbirt, Taiba Zornitzki

**Affiliations:** 1 Internal Medicine Department B, Kaplan Medical Center, Hebrew University Medical School, Rehovot 76100, Israel; 2 Department of Clinical Immunology, Allergy, and AIDS, Kaplan Medical Center, Hebrew University Medical School, Rehovot 76100, Israel; 3 Diabetes, Endocrinology and Metabolic Disease Institute, Kaplan Medical Center, Hebrew University Medical School, Rehovot 76100, Israel; 4 Division of Cardiology, Tel-Aviv Sourasky Medical Center, School of Medicine, Tel-Aviv University, Tel-Aviv 46239, Israel

**Keywords:** hypercalcemia, nontuberculous mycobacterium, HIV, AIDS, *Mycobacterium simiae*

## Abstract

Hypercalcemia represents a rare complication of nontuberculous Mycobacterium (NTM) infections, particularly in individuals with human immunodeficiency virus (HIV) positivity. This systematic review examines NTM infections associated with hypercalcemia, including the presentation of a novel and illustrative case of *Mycobacterium simiae*. A meticulous literature search identified 24 cases relevant to this phenomenon (11 HIV-positive and 13 non-HIV), which were included in the analysis. Key clinical and laboratory findings reveal significant contrasts between HIV-positive and non-HIV patients. In the HIV-positive cohort, hypercalcemia is commonly developed after the initiation of highly active antiretroviral therapy (HAART) or treatment for NTM infections despite severe underlying immunosuppression. Conversely, in the non-HIV group, a spectrum of immunosuppressive conditions, including chronic renal failure and prolonged use of immunosuppressive drugs, was implicated in the pathogenesis of NTM infections with hypercalcemia. Two distinct mechanistic pathways likely underlie this association. In HIV-positive patients, immune restoration following HAART appears to drive granuloma formation and excessive 1,25-dihydroxyvitamin D production. In non-HIV individuals, prolonged immune suppression may facilitate macrophage activation associated with NTM infections, thereby contributing to hypercalcemia. Treatment strategies varied and included bisphosphonates, corticosteroids, and hemodialysis. Notably, bisphosphonates emerged as a safe and effective option in most cases. Antibiotic therapy was deemed unnecessary when hypercalcemia was the sole symptom of NTM infection. This review underscores the importance of recognizing hypercalcemia as a potential complication of NTM infections and tailoring management strategies to the patient’s underlying immunological status.

## 1. Introduction

The evaluation of hypercalcemia starts by measuring parathyroid hormone (PTH) levels in blood. There are two categories: 1-PTH-dependent hypercalcemia and 2-PTH-independent hypercalcemia [1]. Primary hyperparathyroidism (PHPT) is the most common cause in the first group and is due to abnormal, incompletely regulated secretion of PTH from one or more of the four parathyroid glands. Other causes include the use of thiazide diuretics and lithium [2]. The most common etiologies of PTH-independent hypercalcemia include sarcoidosis and tuberculous Mycobacterial disease. Nontuberculous Mycobacteria (NTM) are a rare cause of hypercalcemia, with only a few cases reported in the literature without existing systematic review [3]. The authors conducted a systematic review of NTM-associated hypercalcemia and reported a case of an HIV patient with NTM-induced hypercalcemia treated in our internal medicine department. The goals of the review are to identify the factors associated with hypercalcemia onset, understand its pathophysiology, report the clinical course, and advise treatment of this rare complication.

## 2. Case

A 33-year-old male, recently diagnosed with hepatitis C virus (HCV) and HIV, was admitted to the emergency department (ED) with complaints of fever, somnolence, general weakness, diffuse abdominal pain, nausea, vomiting, and significant weight loss. Antiretroviral therapy (ART) with bictegravir/emtricitabine/tenofovir alafenamide was initiated one month prior to ED presentation. At that time, prophylactic therapy with sulfamethoxazole/trimethoprim was recommended; however, the patient began this treatment only three days before admission without any clinical improvement.

On physical examination, the patient was cachectic, somnolent, and exhibited mild abdominal tenderness. His temperature was 38.5 °C, his blood pressure (BP) was 105/67 mmHg, his pulse was 87 bpm, and his oxygen (O_2_) saturation was 97% in room air. An abdominal computed tomography (CT) scan demonstrated multiple enlarged lymph nodes in the upper abdomen and retroperitoneum.

**Figure 1:**
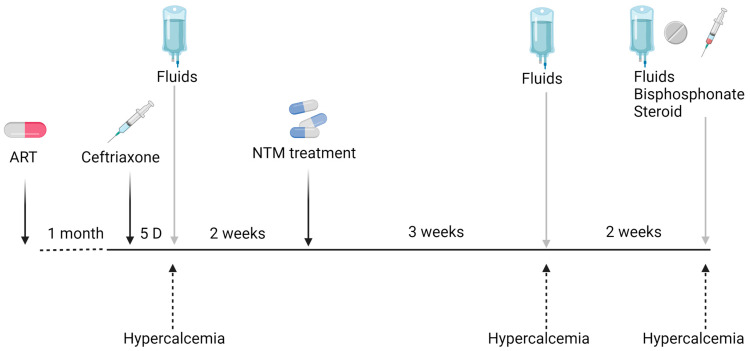
Clinical course of hypercalcemia in a patient with NTM-associated infection.

Laboratory tests showed hemoglobin (Hb) levels of 10.3 g/dL, a low CD4+ count of 30 cells/mm^3^, and an HIV viral load of 1294 copies, while one month before admission the viral load was 8,632,982 copies. His C-reactive protein (CRP) was 27 mg/dL, his serum creatinine was 0.81 mg/dL, his liver enzymes were elevated (AST −159 U/L and ALT 69 U/L), his albumin level was 3.2 g/L, and his corrected calcium was 9.0 mg/dL. Empiric treatment with Ceftriaxone and Acyclovir was initiated due to suspicion of a CNS infection at arrival in the ED. The patient was hospitalized for further investigation. Acyclovir was stopped the following day after a normal lumbar puncture. On the fifth day of hospitalization, the patient’s general condition improved, his CRP level decreased to 15.73 mg/dL, and an intra-abdominal lymph node biopsy was performed. On that date, his serum calcium increased to 12.9 mg/dL, and the patient was treated with intravenous fluids, resulting in an improvement in serum calcium. His renal function remained normal throughout the entire hospitalization. The lymph node biopsy was positive using Ziehl–Neelsen stain, and PAS diastase highlighted numerous intracellular microorganisms consistent with Mycobacterium infection. Empiric treatment was initiated with Clarithromycine, Ethambutol, and Rifabutin. *Mycobacterium simiae* was identified by culture, and the treatment was changed to Clarithromycin, Sulfamethoxazole/Trimethoprim, and Moxifloxacin. The CRP value dropped to 5 mg/dL. The HIV treatment was changed to Dolutegravir/Emtricitabine/Tenofovir. The patient was discharged after a three-week hospitalization without fever and in good general condition (Figure 1). Three weeks later, the patient was readmitted to the hospital due to a significant deterioration in renal function, with his serum creatinine increasing to 3.94 mg/dL, his serum urea was 85 mg/dL, and his corrected calcium was 13.4 mg/dL. His renal failure resulted from diuresis secondary to hypercalcemia. The other laboratory test results were as follows: CD4: +25/mm^3^, CRP: 1.3 mg/dL, PTH: 4.0 pg/mL, angiotensin-converting enzyme (ACE): 172 U/L, vitamin 25(OH)D: 50 nmol/L, 1,25(OH)_2_D: 86.7 pmol/L, and urinary calcium: 24 mg/dL. During this hospitalization, the patient was treated with fluids for hydration, and nephrotoxic drugs were stopped. Corticosteroids were not initiated due to rapid clinical and laboratory improvement. Two weeks later, during a third hospitalization for hypercalcemia and renal failure (due to dehydration), the patient was treated with intravenous fluids, intravenous bisphosphonate-zoledronic acid (5 mg) after improvements in his renal function, and 20 mg prednisone for three days. Hypercalcemia was completely resolved with the normalization of renal function.

One year later, his serum calcium and creatinine were still within the normal range. Unfortunately, the patient died in another hospital due to hepatic failure. Throughout the follow-up, the compliance with HIV treatment was very low, with CD4 values around 20–30 cells/mm^3^, and the viral load was never undetectable. No HIV drug resistance was detected. The patient did not receive treatment against HCV, probably because of his non-compliance.

## 3. Materials and Methods

This study conducted a systematic search for reported cases of NTM-associated hypercalcemia in the English-language literature. The search spanned multiple databases, including PubMed, Embase, Scopus, and Web of Science, covering the period from 1 January 1994 to 28 November 2022. The keywords used were “hypercalcemia and non-tuberculous Mycobacteria/atypical Mycobacteria”. Full data regarding search words and research articles can be found in the Supplementary File (Supplement S1). The identified cases were categorized based on HIV status into two groups: NTM-associated hypercalcemia in HIV-positive patients and HIV-negative patients. Our thorough search yielded a total of 125 articles (Supplement S1) across the four databases. Articles were selected based on the title and the abstract. Some articles were found in several databases. After careful review, twenty-three published cases dealing with NTM and hypercalcemia were included in this comprehensive review. Articles were excluded if an abstract or full-text article was not available.

## 4. Results

The clinical and laboratory data of twenty-four patients with NTM-associated hypercalcemia, including our patient, are presented in Table 1 [4,5,6,7,8,9,10,11,12] and Table 2 [13,14,15,16,17,18,19,20,21,22,23,24,25] for patients with HIV infection- and non-HIV-related immunodeficiency, respectively. The HIV patients were younger compared to non-HIV patients, with a median age of 37 ± 9.5 years vs. 61 ± 13.9 years (*p*-value < 0.01), respectively. In both groups, most patients were male: 10 (90%) in the HIV group and 9 (69%) in the non-HIV group. The median corrected calcium level was 13.9 ± 3.42 mg/dL and 12.9 ± 8.97 mg/dL in the HIV and non-HIV groups, respectively. Vitamin 1,25(OH)_2_D analysis was performed in 10/11 and 8/13 of the HIV and non-HIV patients, respectively. The levels were normal or elevated in all of them (Table 1 and Table 2), except in one case [21]. Vitamin 25(OH)D levels were evaluated in 5 HIV patients and 13 non-HIV patients. The levels were in the normal low reference range (Table 1 and Table 2). PTH levels were reported in 23 (96%) patients. PTH levels in both groups were below the normal reference range except for two chronic renal failure patients with normal low PTH levels [16,22].

In the HIV group, the most common NTM was *Mycobacterium avium complex* (MAC), identified in nine (81%) patients. Other Mycobacteria identified in two HIV patients, including our case, were *Mycobacterium abscessus* [4] and *Mycobacterium simiae*. The CD4 levels were very low in all HIV patients, with a median of 30 mm^3^/mL (range: 1–55). In non-HIV patients, MAC was identified in only 3/13 (23%) patients. Other NTMs included *Mycobacterium abscessus*, *fortuitum*, *marinum*, *microti*, *haemophilum*, *bovis*, *chimaera*, and *kansasii*.

The causes of immunodeficiency in non-HIV patients included adult-onset acquired immunodeficiency syndrome, immunosuppressive drug treatment in four patients, two patients suffering from chronic obstructive lung disease, and four suffering from chronic renal failure (Table 2).

In the HIV group, most patients developed hypercalcemia after a period of four weeks (median: 4 and range: 2–6) with antiretroviral therapy (ART) and/or NTM treatment initiation. In the non-HIV group, the precipitating factor was identified in 50% of the patients. NTM therapy initiation and/or a reduction in immunosuppressant drugs were found as precipitating factors in most cases (Table 2).

Treatment for hypercalcemia included intravenous fluids in almost all patients in both groups. In HIV patients, seven were treated with bisphosphonates, five received steroids, and one patient underwent urgent hemodialysis due to acute renal failure. In non-HIV patients, five received bisphosphonates, six received steroids, and two underwent dialysis, with one of them being after renal transplantation (Table 1 and Table 2).

Follow-up data on the course of hypercalcemia were available for 15 patients. Unfortunately, four patients passed away: one due to hypercalcemia associated with NTM, one from a systemic BCG infection, one from bacterial pneumonia two months after the hypercalcemia episode, and our patient, who died from liver failure.

## 5. Discussion

The current study aimed to examine the association between NTM infections and hypercalcemia in patients both with and without HIV. Our findings provide novel insights into this rare condition, outlined as follows: First, hypercalcemia may manifest after the initiation of antibiotic/ART treatment or due to a reduction in immunosuppressive drugs. When drug-related, the duration of hypercalcemia is typically shorter (approximately one month), whereas it tends to persist longer when associated with recovery from relative immune suppression. Second, the impact of antibiotic therapy on the course of hypercalcemia depends on the stage of the underlying pathophysiology. Importantly, steroids and antibiotics may not always be required for effective management. The current study will begin by reviewing the pathophysiology of hypercalcemia, explore its diverse causes, and describe its progression through three distinct phases. Lastly, the current study will address additional findings related to hypercalcemia, such as vitamin D levels, PTH, and treatment options.

The diagnostic workup for hypercalcemia typically begins with measuring PTH levels. PTH-dependent hypercalcemia, characterized by normal or elevated PTH levels, includes conditions such as primary and tertiary hyperparathyroidism, lithium-induced hypercalcemia, multiple endocrine neoplasia (MEN) disorders, and familial hypocalciuric hypercalcemia [3,26,27]. Conversely, PTH-independent hypercalcemia, which is associated with suppressed PTH levels, may arise from malignancy, prolonged immobilization, hyperthyroidism, vitamin D intoxication, or granulomatous disease [28]. Hypercalcemia associated with elevated levels of 1,25(OH)_2_D has been documented in patients with granulomatous diseases, such as sarcoidosis, tuberculosis, and certain systemic fungal infections. This phenomenon is attributed to the upregulation of 1-alpha-hydroxylase by activated macrophages, which increases the conversion of 25(OH)D to 1,25(OH)_2_D. A recently published review summarizes several rare causes of hypercalcemia associated with granuloma formation, non-granulomatous macrophage infiltration, or increased expression of 1-alpha-hydroxylase by histiocytes. NTM is recognized as a rare cause of hypercalcemia and has only been described in a few case reports [3].

Refractory hypercalcemia is a condition characterized by persistently high serum calcium levels that do not respond adequately to standard treatments. This condition is often associated with malignancies and can be life-threatening if not managed effectively. Refractory hypercalcemia typically occurs when serum calcium levels remain elevated despite aggressive treatment, calcium levels quickly rebound after initial treatment, or conventional therapies fail to maintain normal calcium levels [29]. In contrast, the normal mycobacterial immune response does not typically involve hypercalcemia. While granuloma formation is a key feature of the mycobacterial immune response, it does not directly cause hypercalcemia.

**Figure 2:**
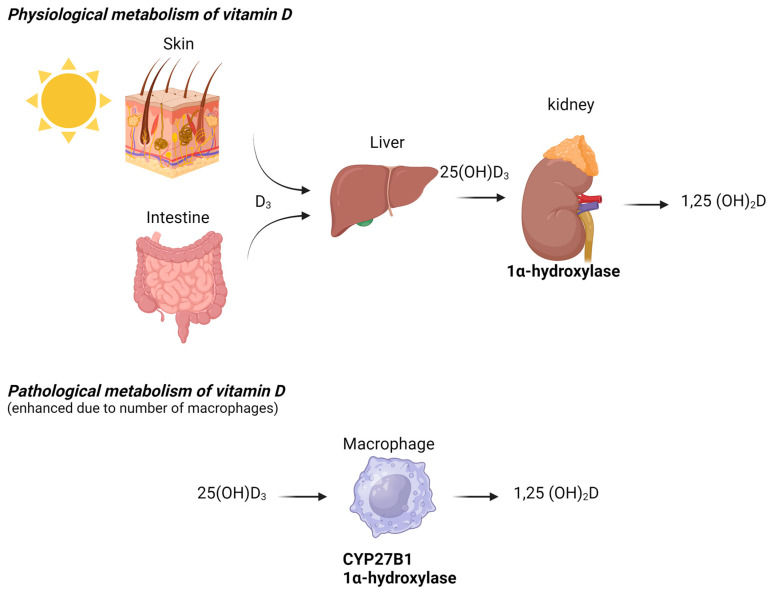
Physiological and pathological metabolism of vitamin D.

In HIV patients, hypercalcemia occurs following immune reconstitution. The beneficial effects of antiretroviral therapy (ART) restore CD4+ T lymphocytes and normalize the immune response. However, adverse clinical phenomena, including the paradoxical worsening of treated opportunistic infections or previously subclinical untreated infections, may develop during the initial months or even years of ART. These atypical presentations, recognized as inflammatory reactions against dormant opportunistic pathogens following CD4 increases with ART (even when CD4 levels remain low), are collectively known as immune reconstitution inflammatory syndrome (IRIS), which is well-documented in HIV-infected populations [30]. In some HIV-infected patients, NTM-associated hypercalcemia can be explained by the restoration of the immune response after the initiation of HIV treatment, which promotes granuloma formation and the production of 1,25(OH)_2_D by activated macrophages (Figure 2). All HIV patients in our review who developed hypercalcemia had severe immunodeficiency, marked by significantly low CD4 counts, which is a known risk factor for IRIS [30]. IRIS, however, has rarely been detected in immunocompromised HIV-negative patients. Several cases have been reported after the initiation of tuberculosis treatment [31] or during solid organ transplantation [32].

In our specific case, the initiation of anti-HIV therapy one month prior to the onset of hypercalcemia contributed to a reduction in the high viral load. Despite a low CD4 count (25/mm^3^), the viral load dropped substantially by at least one log10 copies/mL, which is a criterion for IRIS [33]. During the patient’s initial hospitalization, clinical and laboratory improvement was observed with fluid therapy and Ceftriaxone treatment. Since *Mycobacterium simiae* is resistant to Ceftriaxone, the mechanism by which this antibiotic contributed to the patient’s clinical improvement remains unclear. It is possible that an undiagnosed infection was treated. However, despite multiple repeated blood, sputum, and urine cultures, no other pathogen was isolated.

Another potential cause of hypercalcemia associated with NTM infection could be the treatment itself [4]. It is plausible that antibiotic therapy plays a role in freeing the immune system from suppression or disrupting the evasion strategies employed by NTM bacteria. Certain NTM species, such as *Mycobacterium avium* and *Mycobacterium abscessus,* and particularly those linked to chronic lung infections, exhibit immunomodulatory properties by suppressing immune cell function and inhibiting cytokine production, contributing to persistent infections [34,35]. NTM has a dual effect on the immune system. While these mycobacteria exert immunosuppressive effects, they also stimulate immune responses, including macrophage activation and granuloma formation. The balance between immune activation and suppression is influenced by factors such as the specific mycobacterial species, the host’s immune status, and the stage of infection. In the context of mycobacterial infections, antibiotics play a crucial role in shifting the balance from immunosuppression to immune activation. By reducing the pathogen burden, antibiotics help restore normal immune function and promote immune activation.

In non-HIV patients, only a few reports have suggested that NTM treatment induces hypercalcemia [14,15,19]. Additionally, Parsons et al. described a patient with end-stage renal disease and NTM-induced hypercalcemia caused by the infection itself. In this case, NTM treatment was not required because the patient had no pulmonary symptoms [20]. Among non-HIV patients, hypercalcemia may also appear following immune restoration. It could be an early sign of immunodeficiency in conditions such as chronic kidney disease [36] or COPD [37]. Notably, patients in the non-HIV group were older than those in the HIV group. Age served as an additional risk factor for hypercalcemia in non-HIV patients, likely due to multiple comorbidities or their treatments.

**Figure 3:**
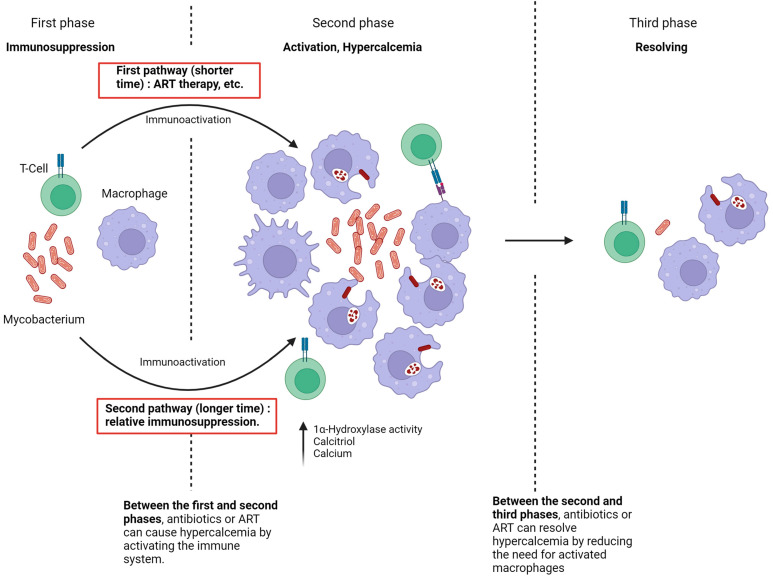
Pathophysiology of NTM-associated hypercalcemia.

The current study hypothesizes that the pathophysiology of hypercalcemia development in immunosuppressed patients infected with NTM consists of three key phases (Figure 3). In the first phase, there is complete immune suppression. The second phase, characterized by the onset of hypercalcemia, occurs when the immune system is no longer suppressed, leading to macrophage activation and significant production of 1,25(OH)_2_D.

Two pathways can lead to the second phase.

The first pathway involves immune suppression, as seen in untreated HIV patients. During this period, NTM spreads within the patient. When the immune system begins to recover, either through ART therapy in previously untreated HIV patients and the initiation of NTM treatment or following the reduction or discontinuation of immunosuppressive drugs [18,22], the large number of atypical Mycobacteria, combined with the rapid restoration of immune function, facilitates granuloma formation and substantial production of 1,25(OH)2D [6]. This typically results in hypercalcemia within a short timeframe, usually around four weeks.

The second pathway to the development of hypercalcemia occurs when the patient experiences relative but sufficient immunosuppression, as seen in conditions like CKD or with suboptimal NTM therapy [8]. In these cases, the immune system is unable to eradicate NTM, leading to prolonged macrophage stimulation and a gradual increase in 1,25(OH)_2_D production. The factors leading to hypercalcemia in these cases may develop slowly and remain undetected.

In the third phase of hypercalcemia, the immune system successfully eradicates the Mycobacterium, resulting in the resolution of hypercalcemia. Thus, if NTM treatment is initiated during the first phase, it may induce hypercalcemia by enhancing immune activation. Conversely, initiating NTM treatment in the second phase leads to the resolution of hypercalcemia due to the elimination of the NTM infection.

In summary, the granulomatous inflammatory response drives hypercalcemia in Mycobacterial infections, and either ART or a paradoxical increase in inflammation after reducing the bacterial load may result in an increase in active vitamin D.

In the case report, the biopsy revealed cores of fibrous tissue containing poorly defined granulomas and numerous macrophages interspersed with small numbers of lymphocytes and neutrophils. The predominance of T cells among the lymphocytes suggests significant macrophage activation.

Remnants of dead mycobacteria can indeed induce granulomatous inflammation, as evidenced by various studies. Mycobacteria exploit the granuloma environment, where dead macrophages can be phagocytosed by newly recruited immune cells, promoting further granuloma expansion and inflammation [38]. This process is crucial as it allows mycobacteria to persist within the host, leading to chronic inflammation and potential disease progression [39]. Moreover, the presence of mycobacterial components, such as exosomes containing mycobacterial proteins, can activate immune responses, sustaining granulomatous inflammation even after the initial infection has been cleared [40]. Additionally, the dynamics of granuloma formation are influenced by the interactions between mycobacteria and various immune cells, including dendritic cells and macrophages, which can perpetuate the inflammatory response [41,42]. Thus, the remnants of dead mycobacteria play a significant role in maintaining the inflammatory milieu characteristic of granulomatous diseases.

Various factors contribute to the lower prevalence of hypercalcemia in NTM compared to tuberculosis. In NTM, the immune response is comparatively weaker, influenced, in part, by differences in the bacterial species causing the infection, as well as variations in the host’s immune response [43,44].

The present study identified 24 reported cases in the literature review. With the exception of a single case [21], the levels of vitamin 1,25(OH)_2_D were either within or above the normal reference range. Vitamin 25(OH)D levels were normal in most cases. This phenomenon is explained by the increased production of vitamin 1,25(OH)_2_D from vitamin 25(OH)D in granulomatous or non-granulomatous macrophage infiltration. Schrayef et al. documented a case of hypercalcemia following treatment with vitamin D and calcium supplements, highlighting how, in the context of NTM, such supplements may contribute to hypercalcemia by accelerating the process of dysregulation [5,13]. It is important to note that studies indicate that dysregulated calcium homeostasis can lead to impaired macrophage responses, resulting in decreased efficacy against *Mycobacterium tuberculosis* [45,46]. A similar mechanism may exist in NTM. As said earlier, studies have highlighted the regulation of the enzyme 1-alpha-hydroxylase in macrophages, particularly in the context of immune responses and disease states. Activated macrophages can produce 1,25-dihydroxyvitamin D (1,25(OH)_2_D) through the expression of 1-alpha-hydroxylase, which can contribute to hypercalcemia in granulomatous diseases [3]. This enzyme’s activity is influenced by various factors, including inflammatory cytokines and vitamin D levels, suggesting a complex interplay between immune activation and vitamin D metabolism [47]. Moreover, the regulation of 1-alpha-hydroxylase in macrophages is critical for their functional polarization, particularly in the context of infections like tuberculosis, where it modulates the production of antimicrobial peptides [48]. The expression of this enzyme is also linked to the metabolic reprogramming of macrophages during inflammatory responses, indicating its potential role in shaping macrophage function and immune outcomes [49]. Research indicates that the expression of 1-alpha-hydroxylase in macrophages can be modulated by inflammatory stimuli. Furthermore, the presence of pro-inflammatory cytokines, such as TNF-alpha and IL-1beta, can enhance the expression of 1-alpha-hydroxylase in macrophages, indicating a link between inflammation and vitamin D metabolism [50]. In addition to cytokine regulation, the expression of 1-alpha-hydroxylase in macrophages is also influenced by the local microenvironment. For example, hypoxic conditions can activate HIF-1α, which, in turn, regulates the expression of various genes, including those involved in the inflammatory response and potentially in the regulation of 1-alpha-hydroxylase [51,52]. Moreover, the interaction between macrophages and other immune cells can further modulate the expression of this enzyme, highlighting the importance of cellular communication in immune responses [53]. Interestingly, the regulation of 1-alpha-hydroxylase is not solely dependent on pro-inflammatory signals. Studies have shown that glucocorticoids can inhibit the expression of this enzyme, suggesting that the balance between pro-inflammatory and anti-inflammatory signals is critical for its regulation [54].

PTH levels were low across the entire study group, except in two patients with chronic kidney disease (CKD) [16,22].

A clinical clue suggesting hypercalcemia due to NTM is acute renal failure. Typically, acute renal failure is accompanied by hypocalcemia, but the presence of hypercalcemia suggests a systemic condition causing the renal failure [55].

It is noteworthy that nearly all HIV patients had MAC infections, while in patients with relative immunosuppression, the spectrum of infections appeared more diverse. This observation suggests that the infection may be linked to high or repeated exposure to NTM in the patient’s environment [14,15,17,22,25].

Patients in both groups were treated with fluid therapy, bisphosphonates, calcitonin, steroids, and occasionally hemodialysis. Although hypercalcemia levels were higher in the HIV group, the treatment decisions were similar in both groups. Bisphosphonate therapy was administered to seven patients in the HIV group and six patients in the non-HIV group, suggesting its effectiveness and safety. Only one-third of the patients received steroid treatment: three in the HIV group and six in the non-HIV group. Thus, the initiation of steroids in patients with immunosuppression and active NTM infection is not immediately necessary but should be contingent upon the responsiveness of hypercalcemia to treatment or the presence of other indications, such as IRIS. In cases of severe symptomatic hypercalcemia, hemodialysis should be considered. Collectively, these cases indicate that when initiating treatment with vitamin D and/or calcium in immunosuppressed patients, vigilant monitoring is imperative. If there are no NTM-related symptoms apart from hypercalcemia, antibiotic therapy may be avoidable. Steroid treatments can suppress the immune system, which could potentially impact the body’s ability to fight infections and form effective granulomas [56]. While steroids are often used to treat various conditions, their immunosuppressive effects can have unintended consequences [57]. Targeting hypercalcemia alone without considering the broader immune implications could lead to inadequate treatment of conditions like NTM infections or result in weaker granuloma formation. This highlights the importance of a comprehensive approach to treatment that considers both the intended effects and potential side effects of steroid therapy. Granuloma formation is a hallmark of chronic inflammatory and infectious diseases, playing a crucial role in pathogen containment and immune regulation. However, its persistence or dysregulation can contribute to disease progression and relapse. Bisphosphonates, widely used for bone disorders, have emerging immunomodulatory effects, potentially influencing granuloma stability and immune responses [56]. Likewise, other immunomodulatory therapies may alter granuloma architecture and function, affecting disease resolution or recurrence [58]. Understanding how these interventions impact granuloma dynamics and relapse rates is essential for optimizing treatment strategies, minimizing disease recurrence, and improving long-term patient outcomes. Further investigation could refine therapeutic approaches by balancing immune control and disease resolution while mitigating adverse effects. Individualized treatment should be guided by the severity and recurrence of hypercalcemia symptoms, manifestations of NTM infection, vitamin D levels, and the potential side effects of prescribed medications.

This review highlights the importance of screening patients for Mycobacterial disease before starting ART [59].

In summary, to the best of our knowledge, this is the first complete review of NTM-associated hypercalcemia with a description of a unique case of hypercalcemia caused by *Mycobacterium simiae*. Hypercalcemia, especially in HIV patients, may be caused by several etiologies, including NTM infection. Patients with NTM and hypercalcemia typically presented with weight loss, weakness, and somnolence. Hypercalcemia can appear shortly either after the initiation of ART/NTM treatment or a reduction in immunosuppressive drugs (short-term) or following recovery from relative immune suppression (long-term). In Mycobacterial infections, the granulomatous inflammatory response drives hypercalcemia, and either ART or a paradoxical increase in inflammation after reducing the bacterial load may result in elevated active vitamin D levels. A positive culture/staining of NTM, acute renal failure, low PTH, or normal/high 1,25(OH)_2_D suggests hypercalcemia etiology. In non-HIV patients, hypercalcemia caused by NTM suggests an immunodeficient state. The treatment approach for hypercalcemia involves fluid administration, bisphosphonates, and calcitonin. Steroid therapy should be considered if conventional therapy is ineffective or if there is an additional indication for steroid treatment. The impact of antibiotic therapy on hypercalcemia depends on the stage of pathophysiology. If there are no NTM-related symptoms, antibiotic therapy might be unnecessary. Further studies are required to fully comprehend the dual mechanism of NTM-induced hypercalcemia and establish therapeutic guidelines.

While case reports offer valuable insights, particularly for rare conditions, it is essential to acknowledge these biases and limitations when interpreting the findings and drawing conclusions. This systematic review relies solely on reported cases, which introduces several biases, including publication bias, selection bias, reporting bias, confirmation bias, and citation bias. Additionally, there are several limitations, such as lack of standardization, small sample size, absence of a control group, limited follow-up data, incomplete information, and subjectivity in interpretation.

## 6. Conclusions

Hypercalcemia, characterized by elevated calcium levels in the blood, has been associated with various infectious diseases, including those caused by NTM. NTM infections, particularly those caused by species such as MAC and *Mycobacterium abscessus*, have been increasingly recognized as significant health concerns, especially in immunocompromised individuals.

One proposed mechanism is the role of vitamin D metabolism. NTM can influence calcium metabolism through the production of vitamin D metabolites, which can lead to hypercalcemia. Moreover, the clinical manifestations of hypercalcemia in the context of NTM infections can complicate diagnosis and management. Symptoms such as fatigue, confusion, and renal impairment may overlap with those of mycobacterial infections, making it challenging to discern the underlying cause of hypercalcemia. Therefore, a thorough evaluation of calcium levels and metabolic status is essential in patients presenting with NTM infections, particularly in those with risk factors for hypercalcemia.

## Figures and Tables

**Table 1:** Description of HIV patients with hypercalcemia and NTM.

	NTM	Calcium Level (mg/dL)	Precipitating Factors of Hypercalcemia	Time Between Factors and Hypercalcemia	1,25 (OH)_2_ (pg/mL)	25 (OH) (ng/mL)	PTH (pg/mL)	ACE (U/I)	Treatment of Hypercalcemia
1. Abdulfattah et al. [4]	*M. abscessus*	16.49	ART	4 weeks	44.1	56	4		Fluid, pamidronate, calcitonin
2. Shrayyef et al. [5]	MAI	13.7	VIT D 25 (OH) treatment/ ART/NTM t	3 weeks	53	42	<2.5		
3. Ayoubieh et al. [6]	MAI	14	NTMt	4 weeks		9	6		Fluid, pamidronate, calcitonin
4. Delahunt et al. [7]	MAI	14.99	NTMt	Unknown	142		<0.3		Fluid, furosemide pamidronate, steroid
5. Choudhary et al. [8]	MAI	12.87	ART/higher NTMt	24 weeks	High				Steroid
6. Playford et al. [9]	*M. avium*	14.63	Higher NTMt	1 week	54		Low		Oral fluid, pamidronate
7. Playford et al. [9]	*M. avium*	13.27	NTMt	4 weeks	83.3		Low		Fluid, pamidronate
8. Tsao et al. [10]	*M. avium*	Ionized 8.1	ART	6 weeks	88.7		3.6		Hemodialysis, steroid
9. Awasty et al. [11]	*M. avium*	14.1	ART	3 weeks	86.7	54.1	4		Calcitonin, pamidronate, steroid, HAART discontinuation
10. Ismailova et al. [12]	MAI	14.5	ART	Not reported	Normal		Normal		Fluid, furosemide, calcitonin, pamidronate
11. Cohen et al.	*M. simiae*	12.4	Antibiotic/NTMt/ART	3 weeks	86.7	50	4	172	Fluid, zoledronic acid, steroid

**Table 2:** Description of non-HIV patients with hypercalcemia and NTM.

	NTM	Calcium Level (mg/dL)	Precipitating Factor of Hypercalcemia	Time Between Factor and Hypercalcemia	1,25 (OH)_2_ (pg/mL)	25 (OH) (ng/mL)	PTH (pg/mL)	ACE U/I	Immune Deficiency Cause	Treatment of Hypercalcemia
1. Kuthiah et al. [13]	*M. abcessus* and *fortuitum*	11.22	TB t	Unknown		23	1	83	Adult-onset immune deficiency	Fluids, steroid, zoledronic acid
2. Moloney et al. [14]	*M. bovis*	12.9	NTM t	Unknown/2 months		16	5	34	(COPD)	Fluids, zoledronic acid, steroid
3. Nielsen et al. [15]	*M. marinum*	Ionized 7.1	NTM t	4 weeks	18	16.4	0.8	nl	Infliximab	Fluids, pamidronate, hemodialysis
4. Donato et al. [16]	*M. avium complex*	11.9	Unknown	Unknown	76	30	15		(CKD)	Fluids, steroid
5. McGoldrick et al. [17]	*M. microti*	17.75	unknown	unknown		normal	<8		CAH, drugs, low CD8	Fluids, pamidronate,
6. Lin et al. [18]	*M. haemophilum*	19.6	IS stop, calcium	10 weeks	127	18.24	6.8		IS drugs	Hemodialysis
7. Haddad et al. [19]	*M. chimaera*	10.5	NTM t	32 weeks		31	2.7		IRIS	
8. Parsons et al. [20]	MAI	12.8	Unknown	Unknown	40	39	8	71	(CKD)	Steroid
9. Chatterjee et al. [21]	*M. avium complex*	13.6	Unknown	Unknown	11	29	8		(CKD)	Fluids, pamidronate
10. Uijtendaa et al. [22]	*M. marinum*	16.8	NTM t/lower steroid	8 weeks	59.1	27	15.1		(CKD)	Fluids, prednisone
11. Vivatvakin et al. [23]	*M. kansasii*	14.8	Unknown	Unknown		52	4.84		(COPD/liver)	Fluids, calcitonin
12. Flowers et al. [24]	*M. kansasii*	12.6	Unknown	Unknown	25	32.6	1	124	Tacrolimus	Fluids, calcitonin, pamidronate
13. Yashswee et al. [25]	*M. bovis*		BCG therapy	6 months	77	Normal	8.5	55	Unknown	Prednisone

Normal range: Calcium: 8.9–10.3 mg/dL, calcium ionized: 4.5–5.1 mg/dL, 1,25(OH)_2_D: 18–64 pg/mL, 25 OH Vitamin D: 20–60 ng/mL, PTH: 14–65 pg/mL, ACE: 16–85 U/L. TB t: tuberculosis treatment. CAH: chronic active hepatitis. NTM: nontuberculosis Mycobacterium. NTM t: nontuberculosis Mycobacterium therapy. ART: antiretroviral therapy. MAI: *Mycobacterium avium intracellulare*. ID: immunodeficiency. IS: immunosuppressant.

## Supplementary Materials

The following supporting information can be downloaded at: https://www.mdpi.com/article/10.3390/microorganisms13040773/s1.
