# Supplementary material for: Exploring a Rare Association: Systematic Review of Hypercalcemia in Nontuberculous Mycobacterial Infections
Source: Microorganisms. 2025 Mar 28;13(4):773. doi: 10.3390/microorganisms13040773 (PMC12029202; doi:10.3390/microorganisms13040773)

Supplement 1

PUBMED
Date 01/01/1994 to 28/11/2022
((hypercalcemia) AND (mycobacterium)) NOT (tuberculosis)
28 results

EMBASE
Date 1994-2022
Broach search : hypercalcemia AND use atypical mycobacteriosis
52 results

SCOPUS
Date 1994- 2022
((TITLE-ABS-KEY (hypercalcemia) AND TITLE-ABS-KEY (non AND tuberculosis AND mycobacterium))) OR ((TITLE-ABS-KEY (hypercalcemia) AND TITLE-ABS-KEY (atypical AND mycobacterium)))
32 results

Web of science
Date 01/01/1994- 28/11/22
hypercalcemia AND atypical mycobacterium (topic) or hypercalcemia AND non tuberculosis mycobacterium (topic)
13 results

PRISMA FLOW CHART

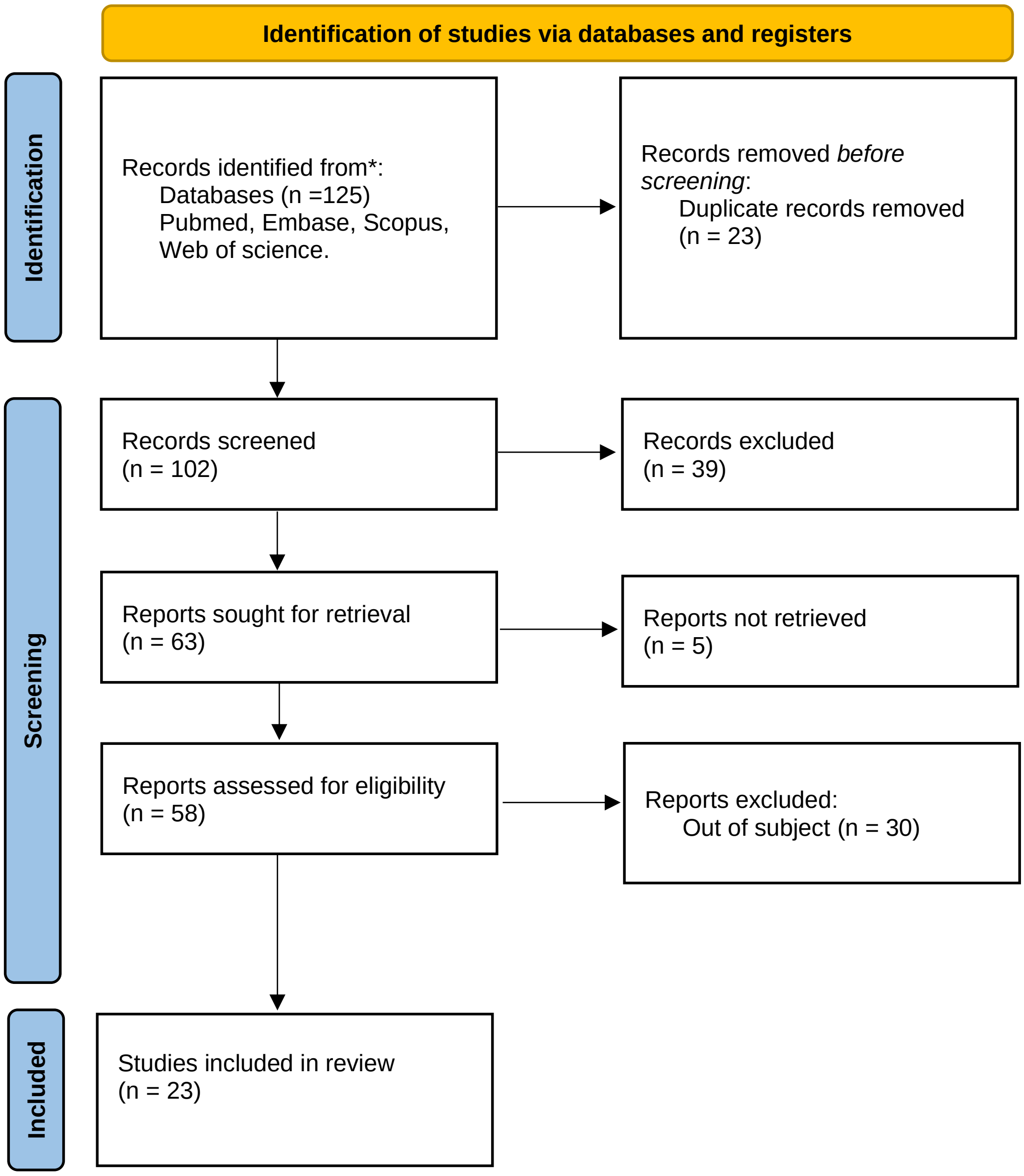

Supplement: Supplementary file 1 [file microorganisms-13-00773-s001.zip › microorganisms-3476382-supplementary.pdf]
